# Fluoroquinolones vs. tetracycline agents combined with rifampicin for periprosthetic joint infections: a comparative study

**DOI:** 10.5194/jbji-11-21-2026

**Published:** 2026-01-12

**Authors:** Benoit Gachet, Olivier Robineau, Maxime Degrendel, Jules Bauer, Bertrand Cappeliez, Emmanuelle Bontemps, Ava Diarra, Pierre Patoz, Eric Beltrand, Eric Senneville, Barthelemy Lafon-Desmurs

**Affiliations:** 1 Infectious Diseases Department, Gustave Dron Hospital, 155 rue du président CotyTourcoing, France; 2 Department of Biostatistics, ULR 2694 METRICS Lille University, Lille, France; 3 INSERM, Institut Pierre Louis d'Epidémiologie et de Santé Publique, UMR-S 1136, AP-HP, Paris, France; 4 Department of Microbiology, Gustave Dron Hospital, 155 rue du président Coty, Tourcoing, France; 5 Department of Orthopaedic Surgery Unit, Gustave Dron Hospital, 155 rue du président Coty, Tourcoing, France

## Abstract

**Objectives**: Periprosthetic joint infections (PJIs) are predominantly caused by gram-positive bacteria. Fluoroquinolones (FQs), combined with rifampicin (RMP), are often used but may be unsuitable due to resistance, side effects, or intolerance. Second-generation tetracyclines (TTCs), such as doxycycline and minocycline, show promise as alternatives. This study compared the efficacy and tolerance of RMP–FQ versus RMP–TTC combinations in PJI treatment. **Methods**: A retrospective study at Tourcoing Hospital, France, reviewed staphylococcal- and streptococcal-related PJI cases treated with RMP–FQ or RMP–TTC from 2013 to 2021. Patients were followed up for 2 years. A Cox regression analysis was used to compare risk of failure. A propensity score using inverse probability of treatment weighting (IPTW) was performed to balance covariates. **Results**: Of 105 patients, 70 received RMP–FQ and 35 RMP–TTC. Infections were mainly monomicrobial (80 %). IPTW-adjusted Cox regression revealed no significant difference in treatment failure between the RMP–FQ and RMP–TTC groups (aHR 0.68; 95 % CI = 0.32–1.4). Subgroup analyses suggested no difference for infections caused by *S. aureus* (HR 1.1; CI 95 % = 0.3–4.0) or coagulase-negative staphylococci (CoNS) (HR 0.54; 95 % CI = 0.1–2.1). Adverse events were similar in both groups (20 % vs. 19 %, p>0.9 ). **Conclusions**: RMP–TTC therapy could be considered a potential therapeutic option for PJIs when FQs cannot be used.

## 1 Introduction

The global population of individuals with orthopedic implants is rising (Sloan et al., 2018) These patients are at risk of various complications, including periprosthetic joint infections (PJIs), which consistently cause considerable morbidity and mortality (Bosco et al., 2023; Zhang et al., 2022). Gram-positive bacteria, particularly *Staphylococcus *spp., are the most frequently implicated microorganisms in these infections (Migliorini et al., 2023; Tande and Patel, 2014; Titécat et al., 2013). The management of PJIs involves both medical and surgical approaches, with several antibiotic regimens recommended (Anon, 2014; Osmon et al., 2013). Bacterial biofilm formation is a key element in the pathophysiology of PJIs (Darouiche, 2001). Due to its maintained activity against biofilm infections (Zimmerli and Sendi, 2019), rifampicin (RMP) is often part of therapeutic regimens when strains are sensitive to this molecule (Osmon et al., 2013). Rifampicin should not be used as monotherapy, and fluoroquinolones (FQs) are generally considered to be first-line companions in these settings (Asseray et al., 2016; Senneville et al., 2011a).

Fluoroquinolones cannot always be used since resistance rates may exceed 15 % for *S. aureus* and 30 % for coagulase-negative staphylococci (CoNS) (Titécat et al., 2013). Additionally, FQ use may be limited by significant side effects, including aortic aneurysm and mitral valvular regurgitation cases during prolonged treatments, as well as metabolic complications, including dysglycemia (Kasanga et al., 2024; Pasternak et al., 2018; Poluzzi et al., 2010; Tomé and Filipe, 2011). Interruption of the rifampicin–levofloxacin combination for intolerance has been reported in 6.4 % of patients treated for a PJI (Vollmer et al., 2021). Moreover, the use of fluoroquinolones contributes to the emergence of multi-resistant bacteria, which may limit their use (Gu et al., 2020). As a result, therapeutic alternatives are crucial, and their evaluation is necessary.

Second-generation tetracycline agents (TTCs), such as doxycycline and minocycline, represent a promising alternative. These bacteriostatic antibiotics inhibit protein synthesis by targeting the 30S ribosome subunit (Joshi, 1997). They have broad-spectrum activity, particularly against Gram-positive bacteria. They have several pharmacokinetic advantages, including good bioavailability, a prolonged half-life ( > 10 h) (Agwuh, 2006), and good bone penetration (Thabit et al., 2019). Clinical data from a limited number of studies on the use of TTCs in the treatment of PJIs show favorable results that need further investigation to fully establish their efficacy and safety (Bart et al., 2020; Cartau et al., 2025; Jang et al., 2024).

This study aimed at comparing the efficacy and tolerance of rifampicin–fluoroquinolone (RMP–FQ) combination versus rifampicin–tetracycline (RMP–TTC) agent combination in PJIs.

## 2 Material and methods

### 2.1 Population, data, and case definition

#### 2.1.1 Study design and population

We conducted a retrospective study in the infectious disease department of Tourcoing Hospital, France. We included adults with PJIs (hip or knee) who underwent debridement and implant retention (DAIR) surgery or one-stage revision, followed by appropriate antibiotic therapy with either RMP–FQ (levofloxacin, ciprofloxacin, or moxifloxacin) or RMP–TTC (doxycycline or minocycline) between January 2013 and December 2021.

#### 2.1.2 Data collection and variables

Patient data were extracted from medical records using a standardized questionnaire. We collected demographic characteristics (e.g., age, gender, weight, body mass index (BMI), renal function, and Charlson score), type of material, number of previous surgeries, microbiological documentation, and the antibiotic regimen received (i.e., either RMP–FQ or RMP–TTC). The planned theoretical follow-up period was 2 years with clinical and biological monitoring.

#### 2.1.3 Definition of cases

PJIs were defined according to the criteria of the European Bone and Joint Infection Society (EBJIS) (McNally et al., 2021). An infection was defined as acute if it occurred within the first 30 d following the implantation of the material. An infection was defined as polymicrobial when two different microorganisms were found in the intraoperative cultures.

### 2.2 Therapeutic approach

#### 2.2.1 Surgical and antibiotic treatment

Surgical interventions included DAIR and one-stage revisions. Broad-spectrum intravenous antibiotic therapy was initiated immediately after intraoperative sampling. Antibiotic therapy was adjusted based on microbiological results after day 5 (or day 14 in the case of associated bacteremia) with either RMP–FQ (levofloxacin, ciprofloxacin, or moxifloxacin) or RMP–TTC (doxycycline or minocycline), depending on microbiological documentation, antibiotic susceptibility testing, known patient allergies or intolerances, and potential drug interactions. The daily dose of RMP was 10 mg per kilogram per day, as already used in previous work by Senneville et al. (2011b) (i.e., 600–900 mg once daily). The doses of levofloxacin, ciprofloxacin, and moxifloxacin were 750 mg once daily, 750 mg twice daily, and 400 mg once daily, respectively. The dose of doxycycline was 200 mg once daily, and the dose of minocycline was 100 mg three times daily (Anon, 2009).

#### 2.2.2 Follow-up and outcome

The primary outcome was failure. Failures included relapse (i.e., recurrence of the infection caused by the same microorganism), superinfection (i.e., caused by a different bacterium not previously identified), or death. Following Escudero-Sanchez et al. (2020), suppressive antibiotic therapy (SAT) was defined as an antibiotic therapy prescribed for an indefinite duration and initiated as a follow-up to an initial treatment lasting 6 to 12 weeks for patients in remission (i.e., lack of clinical symptoms or radiological signs). Indications of SAT were decided at the beginning of management in cases where the infection could not be eradicated, particularly in situations of suboptimal management (DAIR performed after 4 weeks, retention of infected material). Consequently, SAT was not regarded as failure. Adverse events potentially attributable to antibiotics were classified according to the Common Terminology Criteria for Adverse Events (CTCAE) (Common Terminology Criteria for Adverse Events – UpToDate, 2023).

### 2.3 Statistics

#### 2.3.1 Statistical analysis

Baseline characteristics were described using median and interquartile range (IQR) for quantitative variables and number and percentage for qualitative variables. Comparisons between treatment groups were conducted using Fisher's exact test for categorical variables and the Wilcoxon rank-sum test for continuous ones.

Univariate analyses were performed using Cox proportional hazard models. Hazard ratios (HRs) were reported with 95 % confidence intervals (CIs).

#### 2.3.2 Clinical endpoints and propensity score matching

To minimize selection bias due to baseline differences between the treatment groups, we employed a propensity-score-based approach. Each patient treated with RMP–TTC was weighted according to the inverse of their probability of receiving RMP–FQ based on their baseline covariates (i.e., inverse probability of treatment weighting, IPTW). The propensity score was estimated using a non-parsimonious multivariable logistic regression model, which included prespecified baseline covariates: age, Charlson comorbidity index, number of prior surgeries, and type of surgery. Post-weighting, the standardized mean differences between treatment groups were examined for each covariate, with a threshold of 10 % indicating a clinically meaningful imbalance. Rather than using one-to-one matching, which could reduce statistical power by excluding unmatched patients, we opted for IPTW to maximize the utilization of the entire sample and ensure a more efficient adjustment for baseline covariates. To account for immortal time bias, the starting point for survival analysis was defined as the time of the switch to oral therapy. The impact of the treatment on remission was analyzed using a Cox proportional hazard model weighted by IPTW. In a second model, treatment duration (6 weeks vs. 12 weeks) was included as a covariate to account for its potential influence on treatment efficacy.

#### 2.3.3 Subgroup analysis

We conducted subgroup analysis, restricted to patients treated (1) with DAIR or (2) a one-stage revision, as these surgical approaches may yield distinct outcomes. We also compared patients with monomicrobial infections caused by *S. aureus* or CoNS, two common pathogens in PJIs, to assess whether differences in microbiological etiology would impact clinical outcomes. Last, we described the outcome of MRSA-related infections.

#### 2.3.4 Sensitivity analysis

In a cause-specific sensitivity analysis focusing on infection-related failure, deaths adjudicated as unrelated to PJIs were not considered failures, and follow-up was censored at the date of death. This approach assumes that, conditional on covariates, censoring due to unrelated death is non-informative with respect to the hazard of infection-related failure.

All tests were two sided, and p values of ≤ 0.05 were considered statistically significant. Statistical analyses were performed using R 4.0.3.

All data collected for this study from the patient medical folders were entered into a database using anonymous code. Data confidentiality was ensured in accordance with the recommendation of the French commission for data protection (CNIL number: PRO_CNIL_2021_04).

## 3 Results

### 3.1 Population and bacteriology

#### 3.1.1 Study population

A total of 105 patients with a PJI were included in the study and treated at our institution between 2013 and 2021. Of these, 70 patients were treated with the RMP–FQ group, and 35 patients received RMP–TTC. The two groups were comparable in terms of both medical history and comorbidities. There was a significantly higher proportion of cemented joint prostheses in the RMP–TTC group (18 (51 %) vs. 11 (16 %), p<0.001 ). The number of patients who had undergone two or more prosthesis revisions before inclusion appeared to be more frequent in the RMP–TTC group, but this difference did not reach statistical significance (5 (15 %) vs. 4 (6.3 %), p= 0.2). Table 1 summarizes the baseline characteristics of the population.

**Table 1:** Baseline characteristics of patients and description of PJI according to the rifampicin companion regimens.

Characteristic	RMP–TTC	RMP–FQ	p value^2^
	( n= 35)^1^	( n= 70)^1^	
Age (years)	73 (67–84)	72 (64–82)	0.21
Weight (kg)	77 (66–87)	85 (65–99)	0.09
Height (cm)	1.66 (1.62–1.75)	1.70 (1.62–1.76)	0.88
BMI (kg m^−2^)	26 (22–30)	29 (24–34)	0.04
Charlson score	4.00 (3–5)	3.00 (2–4)	0.21
Ongoing cancer treatment	1 (3 %)	1 (1 %)	> 0.99
Diabetes mellitus	7 (20 %)	11 (16 %)	0.58
Chronic kidney disease (GFR < 30 mL min^−1^ 1.73 m^−2^)	2 (6 %)	1 (1 %)	0.26
Rheumatoid arthritis	2 (6 %)	3 (4 %)	> 0.90
Cirrhosis	0 (0 %)	2 (3 %)	0.55
Immunosuppressive therapy	2 (6 %)	4 (6 %)	> 0.90
Type of prothesis			0.49
Knee	19 (54 %)	33 (47 %)	
Hip	16 (46 %)	37 (53 %)	
No. of implant replacements since first implantation			0.20
0	16 (47 %)	40 (63 %)	
1	13 (38 %)	19 (30 %)	
≥ 2	5 (15 %)	4 (6.3 %)	
Cemented prosthesis	18 (51 %)	11 (16 %)	< 0.01
Time to infection (months)	8 (4)	12 (3)	0.80
Concomitant bacteremia	6 (18 %)	13 (19 %)	0.88
CRP at admission (mg L^−1^)	51 (30–118)	63 (33–138)	0.65
Polymicrobial infection	5 (14 %)	16 (23 %)	0.30
Surgical strategy			0.29
DAIR	18 (51 %)	43 (61 %)	
One-stage revision	17 (49 %)	27 (39 %)	
Duration of antibiotic therapy (weeks)			0.29
6	20 (57 %)	46 (68 %)	
12	15 (43 %)	22 (32 %)	
Adverse event	7 (20 %)	13 (19 %)	> 0.90
Early withdrawal due to side effect	2 (6 %)	4 (6 %)	> 0.90
SAT	9 (26 %)	9 (13 %)	0.10

^1^ n (%) and median (Q1, Q3). ^2^ Wilcoxon rank-sum test and Fisher's exact test. RMP–TTC: rifampicin–second-generation tetracycline agent, RMP–FQ: rifampicin–fluoroquinolone, BMI: body mass index, GFR: glomerular filtration rate in CKD-EPI, No: number, CRP: C-reactive protein, DAIR: debridement antibiotics and implant retention, SAT: suppressive antibiotic therapy.

#### 3.1.2 Microbiological findings

Most infections were monomicrobial: 30 (86 %) in the RMP–TTC group and 54 (77 %) in the RMP–FQ group. *Staphylococcus* spp. were the most isolated pathogens, identified in 27 (77 %) of the patients in the RMP–TTC group and in 44 (63 %) of the patients in the RMP–FLQ group. All methicillin-resistant *S. aureus* (MRSA) isolates were found in the RMP–TTC group (8 (66 %)). Coagulase-negative staphylococci were significantly more frequent in the RMP–TTC group (15 (43 %) vs. 11 (16 %), p= 0.002). Details of the microbiological documentation are presented in Table 2.

**Table 2:** Microbiological data.

Characteristic	Cycline	Fluoroquinolone	p value^1^
	( n= 35)^1^	( n= 70)^1^	
Monomicrobial, n (%)	30 (86 %)	54 (77 %)	0.30
*S. aureus*	12 (34 %)	33 (47 %)	0.21
– Methicillin-resistant *S. aureus*	8 (66 %)	0 (0 %)	< 0.01
– Methicillin-susceptible *S. aureus*	4 (33 %)	33 (100 %)	< 0.01
CoNS	15 (43 %)	11 (16 %)	< 0.01
– Methicillin-resistant CoNS	13 (86 %)	4 (36 %)	< 0.01
– Methicillin-susceptible CoNS	2(14 %)	7 (74 %)	0.02
*Streptococcus *spp.	3 (9 %)	10 (14 %)	0.54
Polymicrobial, n (%)	5 (14 %)	16 (23 %)	0.30
Gram-positive cocci and other bacteria^2^	4 (11 %)	10 (14 %)	0.77
Polymicrobial Gram-positive cocci	1 (3 %)	6 (9 %)	0.42
Resistance to FQ, n (%)	24 (69 %)	0 (0 %)	< 0.01

^1^ Fisher's exact test; ^2^ *Acinetobacter pittii, Actinomyces viscosus, Aerococcus viridans, Bacteroides thetaiotaomicron, Citrobacter freundii, Citrobacter koseri, Corynebacterium striatum, Corynebacterium tuberculostearicum, Cutibacterium acnes, Enterobacter cloacae, Escherichia col.*

### 3.2 Therapeutic approach

#### 3.2.1 Surgical and medical treatment

DAIR was performed in 18 (51 %) patients in the RMP–TTC group and 43 (61 %) in the RMP–FQ group. In the RMP–TTC group, the DAIR procedure was more frequently performed after 30 d: 15/18 (83 %) vs. 30/43 (75 %), but without reaching statistical significance ( p= 0.7). The majority of patients treated with RMP–TTC were infected with FQ-resistant strains (69 %), while other patients had contraindications to FQ. The duration of the antibiotic therapy was most often 6 weeks: 20 (57 %) in the RMP–TTC group and 46 (68 %) in the RMP–FQ group. Other patients were treated for 12 weeks. SAT was reported in nine patients in each group, accounting for 26 % in the RMP–TTC group and 13 % in the RMP–FQ group. The median duration of SAT was 74 weeks (IQR 48–166).

#### 3.2.2 Outcome and prognosis

**Figure 1:**
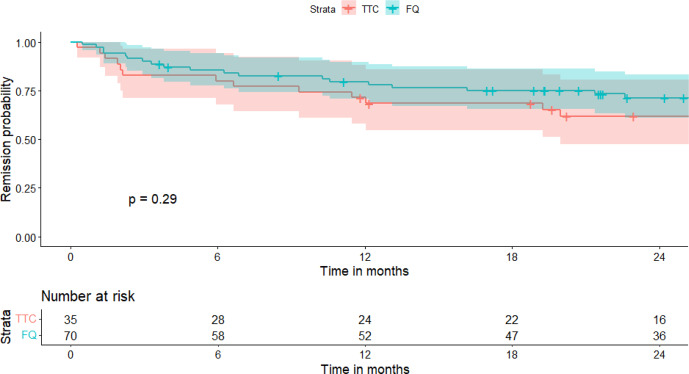
Survival curve in the two treatment groups (univariate analysis). TTC: tetracycline; FQ: fluoroquinolone.

**Figure 2:**
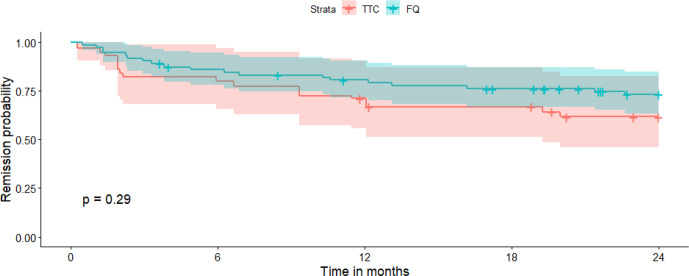
Survival curve in the two treatment groups after propensity score matching. TTC: tetracycline; FQ: fluoroquinolone.

A total of 21 patients (20%) were lost to follow-up before 2 years: 6 (17 %) in the RMP–TTC group and 15 (21 %) in the RMP–FQ group. At 2 years, there were 13/29 (45 %) failures in the RMP–TTC group and 19/55 (35 %) in the RMP–FQ group. We observed 9/22 (41 %) failures in the doxycycline group and 4/7 (57 %) failures in the minocycline group. No statistically significant differences in survival outcomes were observed between RMP–FQ and RMP–TTC: in the univariate analysis, the HR was 0.69 (95 % IC: 0.33–1.39) (Fig. 1), and in the Cox proportional hazard model weighted by IPTW, the aHR was 0.68 (95 % CI: 0.32–1.4) (Fig. 2). After adjustment for the treatment duration, the aHR was 0.65 (95 % CI: 0.3–1.4). Univariate analyses of the variables of the IPTW are shown in Table 3. Details of failure, according to type of prosthesis or type of surgery, are reported in Table 4.

**Table 3:** Univariate Cox regression analyses of parameters included in the propensity score.

Variables	HR (95 % CI)	p
Age, years	1.01 (0.98–1.04)	0.40
Charlson score	1.16 (0.98–1.39)	0.09
≥2 implant replacements since first implantation	1.10 (0.33–3.61)	0.88
One-stage revision	0.60 (0.28–1.27)	0.18

**Table 4:** Detailed cause of failure at 2 years according to prosthesis localization or type of surgery.

Characteristic	Rifampicin–tetracycline			Rifampicin–fluoroquinolone		
Prothesis localization	All	Knee	Hip	All	Knee	Hip
Overall failure^*^	n= 13/29 (45 %)	n= 9/16 (56 %)	n= 4/13 (31 %)	n= 19/55 (35 %)	n= 9/26 (35 %)	n= 10/29 (34 %)
Relapse	1 (8 %)	1 (11 %)	0 (0 %)	2 (11 %)	1 (11 %)	1 (10 %)
Superinfection	6 (46 %)	5 (56 %)	1 (25 %)	13 (68 %)	5 (56 %)	8 (80 %)
Death	6 (46 %)	3 (33 %)	3 (75 %)	4 (21 %)	3 (33 %)	1 (10 %)
Type of surgery	All	DAIR	One-stage revision	All	DAIR	One-stage revision
Overall failure	n= 13/29 (45 %)	n= 6/15 (40 %)	n= 7/14 (50 %)	n= 19/55 (35 %)	n= 16/36 (44 %)	n= 3/19 (15 %)
Relapse	1 (8 %)	1 (17 %)	0 (0 %)	2 (11 %)	1 (6 %)	1 (33 %)
Superinfection	6 (46 %)	3 (50 %)	3 (43 %)	13 (68 %)	11 (69 %)	2 (67 %)
Death	6 (46 %)	2 (33 %)	4 (57 %)	4 (21 %)	4 (25 %)	0 (0 %)

^*^ n (%) – 21 patients had a follow-up of less than 2 years: 6 in the rifampicin–tetracycline group and 15 in the rifampicin–fluoroquinolone group.

Among patients treated with one-stage revision, the RMP–FQ group had a significantly lower risk of failure (HR 0.2, 95 % CI: 0.06–0.88). Of the seven patients who experienced treatment failure in the RMP–TTC group treated with one-stage revision, three developed superinfections and four died (two patients were over 95 years old, and two other deaths were unrelated to the infection). There was no significant difference between the two drug regimens (1) among patients treated with DAIR (HR 0.65, 95 % CI: 0.3–1.4), (2) in the *S. aureus* subgroup (HR 1.1, 95 % CI: 0.3–4.0), and (3) in the CoNS subgroup (HR 0.54, 95 % CI: 0.1–2.1). Details of patients infected with MRSA are reported in Table 5. In the sensitivity analysis in which deaths were excluded from the failure definition, results were consistent with the main analysis. At 2 years, there were 7/29 (24 %) failures in the RMP–TTC group and 15/55 (27 %) in the RMP–FQ group, with no significant difference between treatment groups (HR 1.16, 95 % CI 0.46–2.96, p= 0.75).

**Table 5:** Characteristics and outcome of patients with documented MRSA infection (all patients were in the RMP–TTC group).

Characteristic		Value ( n )	
Type of prosthesis		Hip	Knee
		n= 5 (62 %)	n= 3 (38 %)
Type of surgery		DAIR	One-stage revision
		n= 3 (38 %)	n= 5 (62 %)
Outcome	Remission	5 (62 %)	
	Relapse	0 (0 %)	
	Superinfection	2 (25 %)	
	Death	1 (13 %)	

#### 3.2.3 Adverse effects reported

Adverse events were reported in 7 patients (20 %) in the RMP–TTC group and 13 (19 %) in the RMP–FQ group ( p>0.9 ). Six cases (9 %) of tendinopathy occurred exclusively in the RMP–FQ group. Eight gastrointestinal disorders (nausea, vomiting, or diarrhea) were reported: five (14 %) in the RMP–TTC group and three (4 %) in the RMP–FQ group. Two patients (5.7 %) had to stop the treatment in the RMP–TTC group and four (5.7 %) in the other (see Table 1). None of these adverse events led to hospital admission. No *Clostridium difficile* colitis occurred during treatment and follow-up.

## 4 Discussion

The main result of this survey is that we did not find any significant difference in terms of failure at 2 years when comparing the therapeutic efficacy of RMP–TTC (doxycycline or minocycline) versus RMP–FQ (levofloxacin, ciprofloxacin, moxifloxacin) in patients with PJIs. The rate of adverse effects reported in the two groups was also similar.

In PJIs, the combination of FQ and RMP seems to be the best association in terms of outcome (Cortés-Penfield et al., 2022). However, situations where this combination of treatments cannot be used are common. The spectrum of activity of TTCs corresponds to the microbiology of PJIs. Hamad et al. (2015) studied 134 strains of *Staphylococcus epidermidis* isolated from PJIs: 69 % of them were susceptible to doxycycline (Hamad et al., 2015), and in cases of resistance to doxycycline, other oral TTCs, such as minocycline, may remain effective (Doub et al., 2022). In vitro studies suggest that TTCs have activity against *S. aureus* biofilms, including strains isolated from PJIs (Mandell et al., 2019; Raad et al., 2007).

To date, prioritizing second-line treatments and other companion agents to rifampicin remains challenging (Gachet et al., 2024). TTCs exhibit properties that make them appealing for use in PJIs. The RMP–TTC combination might potentially represent a potent anti-biofilm association. An interaction between rifampicin and doxycycline has been reported, showing decreased doxycycline concentrations (Colmenero et al., 1994; Garraffo et al., 1988). To our knowledge, there are no data on the clinical impact that this interaction might have on treatment efficacy. No known or reported interaction exists between rifampicin and minocycline. As the number of patients treated with minocycline in our cohort was very small, we were unable to assess any potential difference between doxycycline and minocycline. Other interactions are well established, such as those involving clindamycin (Goulenok et al., 2023), linezolid (Bock et al., 2023), or moxifloxacin (Manika et al., 2015) combinations. Most studies on PJIs compared FQs, such as ciprofloxacin and levofloxacin, to other regimens, such as linezolid, clindamycin, and trimethoprim–sulfamethoxazole.

Recently, three studies have published clinical data on TTCs used for PJI treatment. Beldman et al. (2021) reported a significantly higher failure rate when using a companion to rifampicin other than fluoroquinolone or clindamycin: (OR 10.1, 95 % CI, 5.65–18.2). However, these failures were mostly associated with the use of trimethoprim–sulfamethoxazole. Oral TTC agents were used in combination with rifampicin in a few patients ( n= 19; 5.7 %), and the remission rate was similar to that of the RMP–FQ and clindamycin groups (14/19 = 74 %). Bart et al. (2020) evaluated a regimen of vancomycin–minocycline for 4 to 6 weeks followed by oral minocycline monotherapy for 6 to 8 weeks in 34 patients with chronic methicillin-resistant staphylococcal PJI (Bart et al., 2020). There were no significant differences in terms of outcome (relapse, superinfection, or death) compared to the group ( n= 36) treated with vancomycin–rifampicin followed by rifampicin plus another oral companion. Jang et al. (2024) reported positive outcomes with the use of doxycycline in 24 patients who underwent DAIR or had a permanent spacer (Jang et al., 2024). However, the switch to TTCs mostly occurred after 6 weeks and was prolonged as SAT in 13 out of 24 patients.

Most patients included were treated with the DAIR strategy. The failure rate was low and did not differ depending on the treatment regimen in the two groups of interest. This rate was similar to that of a clinical trial and observational study involving a rifampicin-based regimen, mainly a rifampicin–FQ regimen (Beldman et al., 2021). Notably, a significant proportion of these procedures was performed more than 4 weeks after the onset of infection. A third of these patients received SAT after the initial regimen. The timing after which DAIR treatment should be avoided is debated in the literature. This procedure is sometimes chosen by default when it is not possible to change the implant due to technical constraints or the patient's condition. Good results can, however, be achieved, especially if the initial treatment is followed by a suppressive antibiotic therapy (Escudero-Sanchez et al., 2020). The specific timing after which the risk of failure increases is still largely undefined, as are the indications of SAT (Elkins et al., 2019).

Because many deaths in our cohort were judged to be unrelated to PJIs and reflected underlying comorbidities, we performed a cause-specific sensitivity analysis in which such deaths were censored rather than counted as failures. This approach allowed us to focus on infection-related outcomes; importantly, the association between fluoroquinolones versus tetracyclines and treatment failure remained essentially unchanged, suggesting that our main conclusions are not driven by unrelated mortality.

The main strength of our study was to focus our analysis on patients with PJIs treated with DAIR or one-stage revision. This allowed us to maintain comparability in terms of baseline characteristics. Additionally, we used robust statistical methods, including IPTW-adjusted Cox models, to control for confounding and balance the treatment groups.

However, there are several limitations. Notably, 21 patients were lost to follow-up at 2 years but only 6 at 1 year; survival analysis was therefore performed to account for this limitation. Although we employed methods to control for confounding bias, the sample size was relatively small, resulting in limited statistical power and preventing us from drawing firm conclusions regarding the absence of a significant difference between the two therapeutic regimens. Moreover, while we used methods to control for confounding bias, the treatments were not randomly assigned, introducing the potential for unmeasured confounders. For example, while the two groups were comparable regarding almost all comorbidities, RMP–TTC patients had a higher proportion of two or more prior prosthesis revisions. This trend did not reach statistical significance but was consistent with the statistically significant higher presence of cemented prostheses in the RMP–TTC group and a higher risk of re-infection reported with this procedure (Hipfl et al., 2022). Secondly, until 2017, some patients might have received antibiotic therapy for a duration of 6 weeks. This was based on bibliographic data suggesting the non-inferiority of a 6-week duration compared to 12 weeks (Chaussade et al., 2017). After the publication of the DATIPO study, our treatment durations were extended to 12 weeks (Bernard et al., 2021). Of note, the proportion of our patients treated for 6 and 12 weeks was similar between the two groups, and the remission rate was similar between patients treated for 6 weeks and those treated for 12 weeks. Lastly, considering the sample size, we were unable to perform a multivariate analysis of subgroups of interest, such as DAIR and one-stage revision surgery. It seems that TTCs may be less effective in the one-stage revision strategy, but this needs to be confirmed by further studies with a larger sample size.

## 5 Conclusions

Our data suggest that the combination of rifampicin with oral tetracycline agents, such as doxycycline or minocycline, could be considered a potential therapeutic option for PJIs, particularly in cases of resistance or contraindications to fluoroquinolones. Further studies, especially randomized controlled trials, are needed to better prioritize the therapeutic options for PJIs and to optimize fluoroquinolone sparing.

## Data Availability

Data are available upon reasonable request to the corresponding author and if/as approved by the institutional review board. The data are not publicly available due to privacy concerns regarding protected health information.

## Code availability

Code is available upon reasonable request to the corresponding author.
